# Anti-tumor Effect of *Ginkgo biloba* Exocarp Extracts on B16 Melanoma Bearing Mice Involving P I3K/Akt/HIF-1α/VEGF Signaling Pathways

**DOI:** 10.22037/ijpr.2019.1100637

**Published:** 2019

**Authors:** Chen-jie Cao, Ya Su, Jian Sun, Gui-yun Wang, Xiao-qin Jia, Hua-sheng Chen, Ai-hua Xu

**Affiliations:** a *Department of Pharmacology, Medical College of Yangzhou University, Yangzhou, Jiangsu 225001, China. *; b *Department of Combination of Traditional Chinese and Western Medicine, Medical College of Yangzhou University, Yangzhou, Jiangsu, 225001, China. *; c *Department of Pathology, Medical College of Yangzhou University, Yangzhou, Jiangsu, 225001, China. *; d *Jiangsu Co-Innovation Center for Prevention and Control of Important Animal Infectious Diseases and Zoonoses, Yangzhou, Jiangsu, 225009, China.*; 1 CJ. C. *and* Y. S. contributed equally to this work.

**Keywords:** Ginkgo biloba exocarp extracts, Melanoma, Angiogenesis, PI3K/Akt, HIF-1α, VEGF

## Abstract

The objective of this study is to investigate the anti-tumor effect of *Ginkgo biloba* exocarp extracts (GBEE) on B16 melanoma bearing mice and its related molecular mechanisms. The B16-F10 melanoma solid tumor model was established in C_57_BL/6J mice. The tumor-bearing mice were treated with GBEE (50, 100, 200 mg/kg), taking cis-Dichlorodiamineplatinum (Ⅱ) (DDP, 3 mg/kg) as positive control and normal saline (NS) as model control. After 17 days of administration, the transplanted tumors was stripped and weighed, and the inhibition rate was calculated. Quantitative Reverse Transcription Polymerase chain reaction (qRT-PCR), Western Blot and immunohistochemistry were applied to detect mRNA and protein levels of related factors in B16 transplanted tumor tissues. The results indicated that GBEE (50, 100, 200 mg/kg) inhibited the growth of B16 transplanted solid tumor in C_57_BL/6J mice. Meanwhile, it inhibited the expression of CD34 and reduced microvessel density (MVD) in a dose-dependent manner. Moreover, GBEE dose-dependently down-regulated the mRNA and protein levels of hypoxia inducible factor-1α (HIF-1α), vascular endothelial growth factor (VEGF), and vascular endothelial growth factor receptor 2 (VEGFR2). The phosphoinositide 3-kinase (PI3K) and protein kinase B (Akt) proteins were not changed obviously, but the protein levels of p-PI3K and p-Akt were down-regulated. Overall, the inhibitory effect of GBEE on the growth of B16 melanoma transplant tumor in mice is related to inhibiting angiogenesis, and the mechanism involves the regulation of PI3K/Akt/ HIF-lα/VEGF signaling pathway.

## Introduction

Melanoma is a kind of skin tumor resulted from abnormal melanocyte hyperplasia, and it has the characteristics of high malignancy and high mortality (1). The current drugs that are commonly used in clinical treatment of melanoma are east to produce drug resistance and lead to immune-related adverse reactions (2, 3). And thus, it has been an important topic in this field to develop anti-malignant melanoma drugs with good curative effects and few adverse reactions. 

Tumor blood vessels provide the necessary oxygen and nutrients for the growth of tumor cell, and simultaneously, provide access for tumor cells to immerse in blood vessels and transfer to other organs. Therefore, tumor angiogenesis is an important part in tumor growth and metastasis (4). Tumor angiogenesis is a very complex process, and at present, developing anti-tumor drugs that take various aspects of tumor angiogenesis as targets has become a hot research topic in the field of oncology medical treatment (5, 6). 

The fruit of *Ginkgo biloba* L. is also known as Ginkgo nuts which is a traditional Chinese medicine. The treatment of skin diseases and tumors is one of its traditional effects (7). *Ginkgo biloba *exocarp extracts (GBEE) is a composition extracted from *Ginkgo biloba *exocarp taking proteoglycan as effective part (8). Studies have demonstrated that GBEE plays a role in immune promoting (9), and it has inhibitory effects on Lewis lung cancer, sarcoma 180 (S180), liver cancer, gastric cancer,* etc. *(10-12). Moreover, no adverse reactions were observed when applied as a hospital preparation for cancer patients (13, 14). Recent studies of our research group demonstrated that GBEE has an anti-angiogenic effect on Lewis lung cancer, and the mechanism involved inhibiting the expression of VEGF (15). The role of GBEE on melanoma has not been reported. In this study, the B16-F10 transplanted solid tumor model was established in C_57_BL/6J mice to investigate the effects and mechanisms of GBEE on the growth and angiogenesis of melanoma.

## Experimental

*Preparation of GBEE*

Ginkgo nuts samples were obtained from Taixing, Jiangsu Province, and China, identiﬁed by Director of pharmacists Meng Yin in Yangzhou Food and Drug Inspection and Testing Center (Jiangsu Province, China) as the family plant of *Ginkgo biloba* L. The succulent skin was peeled off by hand and then dried up. The dried *Ginkgo biloba* exocarp was sealed at room temperature. GBEE was prepared according to the invention patent in our laboratory. Patent No: CN 201010251050.9 (8). High Performance Liquid Chromatography (HPLC) and Thin Layer Chromatography (TLC) analysis showed that GBEE contained 7 kinds of monosaccharides including mannose, rhamnose, galacturonic acid, glucose, galactose, fructopyranose and arabinose. The HPLC detection also made clear that the protein in GBEE contains 14 kinds of amino acids including aspartic acid, glutamic acid, serine, glycine, threonine, alanine, proline, valine, methionine, isoleucine, leucine, phenylalanine, tryptophan, and lysine. The Infrared Spectroscopy (IR) analysis showed that it contained polysaccharide characteristic peak. The total content of proteoglycan was 66.4% measured by phenol-sulfuric acid method and brilliant blue method. The composition analysis stated that the GBEE did not contain ginkgolic acid, flavonoids and terpene lactones, and the contents of Pb, Cr, Cu, As, and Hg were in accordance with the limited edition requirement of the Chinese Pharmacopoeia. The GBEE voucher specimen was deposited in the pharmacy experimental center in Medical College of Yangzhou University.

*Reagents and Antibodies*

Dulbecco’s modified eagle medium (DMEM) and fetal bovine serum (FBS) were obtained from Gibco (Grand Island, NY, USA). cis-Dichlorodiamineplatinum (II) (DDP) was purchased from Hongda Biotechnology (Beijing, China). Tris base, glycine, and sodium dodecyl sulphate (SDS) were obtained from Biosharp (Anhui, China). Sodium chloride (NaCl), ethanol and other chemical reagents were derived from Sinopharm Chemical Reagent (Beijing, China). Polyvinylidene fluoride (PVDF) membrane was purchased from the Millipore Corporation (Bedford, MA, USA). Immunohistochemistry kit, radio-immunoprecipitation assay (RIPA) lysate, 3,3′-diaminobenzidine (DAB) were obtained from Boster (Wuhan, China). RNA extraction reagent, diethy pyrocarbonate (DEPC), primers, M-MuLV first strand cDNA synthesis kit, 2xSG fast qPCR master mix, anti-CD34, anti-vascular endothelial growth factor (VEGF), Anti-vascular endothelial growth factor receptor 2 (VEGFR2), anti-phosphor-phosphoinositide 3-kinase (p-PI3K), secondary antibodies conjugated to horseradish peroxidase were purchased from BBI (Shanghai, China). Anti-PI3K and anti-hypoxia inducible factor-1α (HIF-1α) were obtained from Wanleibio (Shenyang, China). Anti-phosphor-protein kinase B (p-Akt) and anti-akt were purchased from cell signaling (MA, USA). Anti-GAPDH was obtained from Abcam (Cambridge, England). BeyoECL plus and enhanced BCA protein assay kit were purchased from Beyotime (Shanghai, China).

*Animals*

C_57_BL/6J mice were provided by the Center of Comparative Medicine of Yangzhou University. The mice were female and 6 weeks old, weighing 18-22 g (SPF class). Animal Certiﬁcate: SCXK Su 2012-0004; Animal use license: SYXK Su 2012-0029. Mice had been acclimated on 12-h of light /dark cycle for a week before used. All experiments were conducted in conformity with the Institutional Animal Ethics Committee of Yangzhou University, the National Institutes of Health Guide for Care and Use of Laboratory Animals, and the Principles of Laboratory Animal Care (NIH publication #85−23, revised in 1985).

*Cell culture*

The B16-F10 cells strain was purchased from Shanghai cell bank, Chinese Academy of Sciences. The cells were cultured in DMEM with 10% FBS, incubated at 37 °C in a humidiﬁed atmosphere with 5% CO_2_ (v/v). The cells were subcultured by trypsin digestion method, and the passage time was usually 3 days.

*Inhibition rate of B16 transplanted solid tumor*

The B16-F10 cells cultured *in-vitro* were washed and diluted by Normal saline (NS), preparing the B16 cell suspension. A volume of 0.2 mL of cell suspension was inoculated subcutaneously under the right forelimb armpit of C_57_BL/6J mice, and the cells were passaged in mice. The tumor tissues of tumor-bearing mice was removed and cut, preparing cell suspension by conventional methods. The cell density was adjusted to 1.0 × 10^7 ^cells/mL with NS. The cell suspension was inoculated subcutaneously under the right forelimb armpit of mice, and each mouse was injected 0.2 mL. The next day, the mice were randomly divided into 6 groups, containing 10 mice. The administration groups are as follows, normal control group (without tumor cells) and model control group were given NS at a volume of 0.1 mL/10 g (b.w.) by intragastric gavage (i.g.), once a day for 17 days; the positive drug group was given DDP at a dose of 3 mg/kg (b.w.) by intraperitoneal (i.p.), once every other day for 8 days; the drug group was given GBEE at a dose of 50, 100, and 200 mg /kg (b.w.) by i.g., once a day for 17 days. On the 18^th^ day, the mice were sacrificed and the tumors were completely removed. The blood, adipose, and other tissues were cleaned, and then the tumor mass was weighed. The inhibition rate = (average tumor weight in Model Control group-average tumor weight in Treatment)/average tumor weight in Model Control group × 100%.

*Immunohistochemistry*

The transplanted tumor tissue were fixed with 10% neutral formalin for 24 h, and dehydrated in ethanol. Then, it was embedded in paraffin and sliced. The slices were dried in an incubator, and deparafﬁned in xylene and rehydrated in a gradient of ethanol. The activity of endogenous peroxidase on sections was blocked with 3% H_2_O_2_. The antigens were repaired with microwave ovens. In order to reduce nonspecific reactions, the slices were blocked by 5% BSA at room temperature before being incubated with anti-CD34 for a night at 4 °C. Then, the slices were incubated with horseradish peroxidase-conjugated Goat Anti-Rabbit IgG and dye with DAB and SABC. The slices were finally counterstained with hematoxylin. PBS was used to wash the slices after each step and replace the primary antibody as a negative control. The results of the target protein were obtained under optical microscope. The expression of CD34 protein was located in the cytoplasm of vascular endothelial cells and the positive staining was brown or tan. The MVD was detected by calculating the CD34-positive cells. The expression of HIF-1α protein was located in nucleus and the positive staining was brown. The expression of VEGF protein was located in cytoplasm and the positive staining was brown. Each slice was randomly selected five fields in the high magnification (×200), and the mean integrated optical density (IOD) of CD34, HIF-1α, and VEGF positive chromatin was determined using image analysis software Imagepro plus 6.0.

*Western Blot*

The transplanted tumor tissues preserved in liquid nitrogen were taken out. After rinsed with NS, the tissues were cut into small pieces of 20 mg. The pre-cooled lysate containing protease inhibitor was added, and the homogenizer was used to break the tissues. After centrifugation at 4 °C, the supernatant was stored at -80 °C until use. The protein concentration was determined by BCA assay reagent. The total protein was separated by SDS polyacrylamide gel electrophoresis, and then transferred to PVDF membrane. Five percent BSA was used to seal the membrane at room temperature for 1.5 h. The membrane was incubated with the primary anti-body (Anti-HIF-1α, Anti-VEGF, Anti-VEGFR2, Anti-p-PI3K, Anti-PI3K, Anti-p-Akt, Anti-Akt, and Anti-GAPDH) overnight at 4 °C and then the secondary antibodies conjugated to horseradish peroxidase was added. After 4 h, the fluorescent substrate was prepared and the results were recorded with gel imaging system (BIO-RAD Company, Hercules, CA, USA). The relative expression level of target protein was obtained by using the image analysis software ImageJ to analysis of the gray value, GAPDH as the internal reference.

*qRT-PCR*

The transplanted tumor tissues preserved in liquid nitrogen were taken out. The trizol reagents were added to extract the total RNA. Then, the RNA purity was detected using spectrophotometer. Ten μL total RNA was reverse transcribed into cDNA using 20 μL reverse transcription system with the M-MuLV First Strand cDNA Synthesis Kit. The cDNA was amplified by RT-PCR amplification kit (2xSG Fast qPCR Master Mix). GAPDH was reference gene. The primer sequence of the gene is as follows: VEGF, sense 5′-TGTCTATCAAGGGAGTGTGTGC-3′ and anti-sense 5′-TGGAGTATTTCCGTGA-CCG-3′; VEGFR2, sense 5′-GCGTGATTCTGAGGAAAGG-3′ and anti-sense 5′-ATAAACAGTGG-AGGCTATGTCG-3′; HIF-1α, sense 5′-TGTCTATCAAGGGAGTGTGTGC-3′ and anti-sense 5′-TGGAGTATTTCCGTGACCG-3′.

Each group of samples was repeated three holes, and calculated the average threshold cycle number (Ct). The results of realtime fluores-cence quantitative were analyzed by 2^-^^ΔΔCt^ method, and the value of RQ (relative quantification) was calculated.

*Statistical analysis*

All data in this experiment were sorted and analyzed by SPSS 17.0. The data were realized by unpaired Student’s *t*-test or one-way analysis of variance (ANOVA). Data were expressed as means ± standard error of the mean (SEM). *P *< 0.05 considered to be significant.

## Results

*Inhibitory effect of GBEE on B16 transplanted tumor*

**Figure 1:**
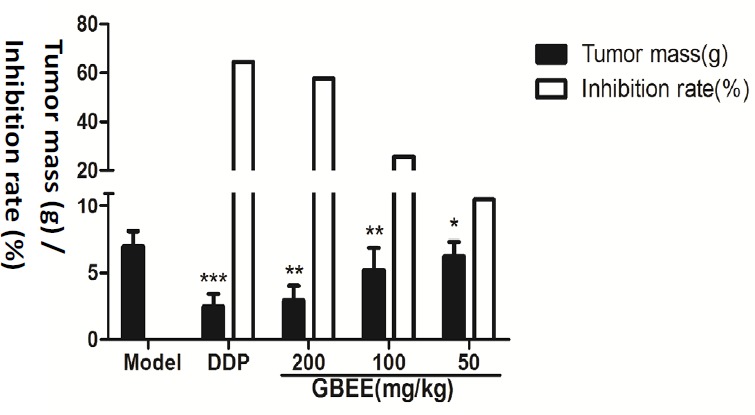
Effects of GBEE on the growth of transplanted tumor. The C57BL/6J mice were injected with B16-F10 cells. Mice with transplantation tumor were treated with normal saline (NS), cis-Dichlorodiamineplatinum (Ⅱ) (DDP, 3 mg/kg), and 50, 100, 200 mg/kg of *Ginkgo biloba *exocarp extracts (GBEE). On the 18th day, mice were sacrificed and the tumor weight was measured, and the tumor mass (g) and inhibition rate (%) was calculated. The tumor mass (g) = average tumor weight in each group. The inhibition rate (%) = (average tumor weight in Model Control group-average tumor weight in Treatment) /average tumor weight in Model Control group × 100%. Data are shown as mean ± SD (n = 10). *P < 0.05, **P < 0.01, ***P < 0.001, *vs. *model control

The tumor weight in GBEE (50,100, 200 mg/kg) groups were decreased in a dose-dependent manner after the mice were given GBEE for 17 days, which were statistically significant compared with the model control group (Figure 1).

*Effect of GBEE on MVD of B16 transplanted tumor*

**Figure 2:**
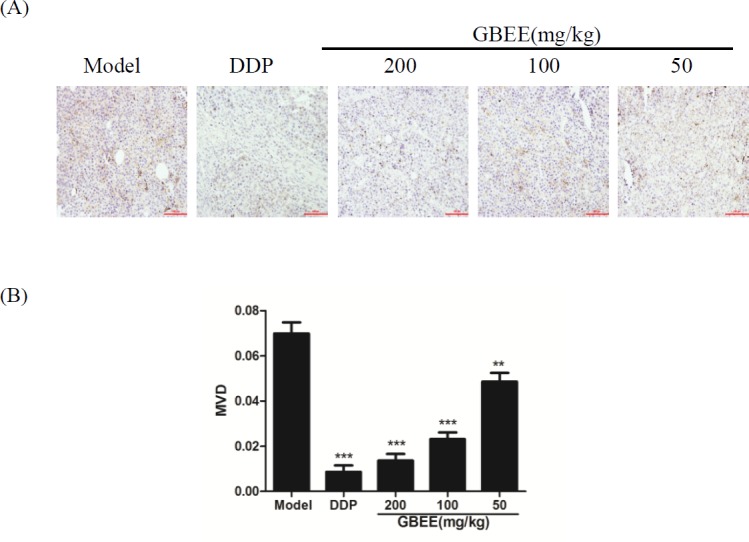
Mice with transplantation tumor were treated with normal saline (NS), cis-Dichlorodiamineplatinum (Ⅱ) (DDP, 3 mg/kg), and 50, 100, 200 mg/kg of *Ginkgo biloba *exocarp extracts (GBEE). On the 18th day, tumor was removed and then tumor tissue section was prepared. (A) Effects of GBEE on the expression of CD34 in B16-F10 transplanted tumor. The level of CD34 in B16 transplanted tumor was determined by immunohistochemistry. The nucleu was dyed as blue, and CD34 was expressed in cytoplasm and was dyed as brown

Compared with the model control group, the expression of CD34 protein in the GBEE treated group was decreased, and the MVD was reduced. Moreover, the effect had dose-effect relationship (Figure 2).

*Effects of GBEE on HIF-1α, VEGF and VEGFR2 in B16 transplanted tumor*

**Figure 3:**
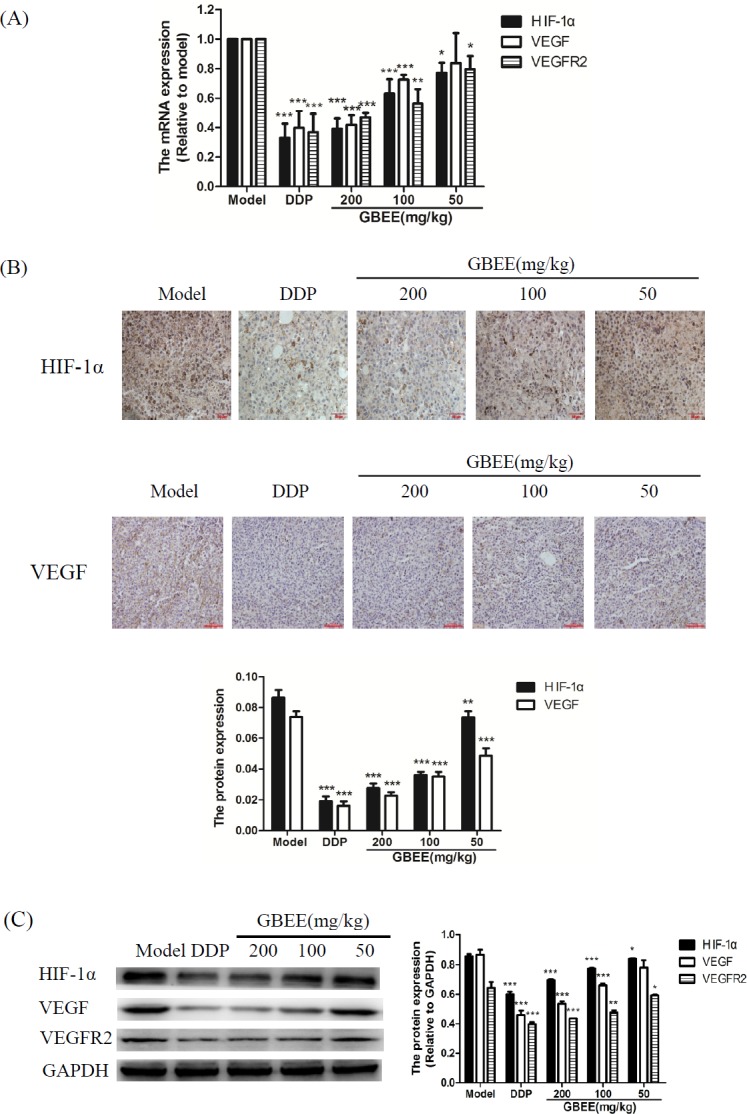
Effects of GBEE on HIF-1α, VEGF and VEGFR2 in B16-F10 transplanted tumor. Mice with transplantation tumor were treated with normal saline (NS), cis-Dichlorodiamineplatinum (Ⅱ) (DDP, 3 mg/kg), and 50, 100, 200 mg/kg of *Ginkgo biloba* exocarp extracts (GBEE). (A) The mRNA expression of HIF-1α, VEGF and VEGFR2 in tumors were analyzed by qRT-PCR. (B) The protein expression of HIF-1α, VEGF and VEGFR2 were determined by immunohistochemistry. HIF-lα accumulated in the nucleus and was dyed as brown. VEGF located in the cytoplasm and was dyed as brown. The histogram was the quantitative result of HIF-1α, VEGF and VEGFR2. (C) The protein expression of HIF-1α, VEGF and VEGFR2 were determined by Western Blot assay. The histogram was the quantitative result of HIF-1α, VEGF and VEGFR2. Data are shown as mean ± SD (n = 3). ^*^P < 0.05, ^**^P < 0.01, ^***^P < 0.001, *vs.* model control

qRT-PCR assay explained that GBEE made the mRNA level of HIF-1α, VEGF and VEGFR2 decreased with the increase of drug dose (Figure 3A). The results of Immunohistochemistry and Western blot showed that GBEE down-regulated the protein expression of HIF-1α, VEGF and VEGFR2 in a dose-dependent manner (Figures 3B and 3C).

*Effects of GBEE on p-PI3K/PI3K and p-Akt/Akt in B16 transplanted tumor*

**Figure 4:**
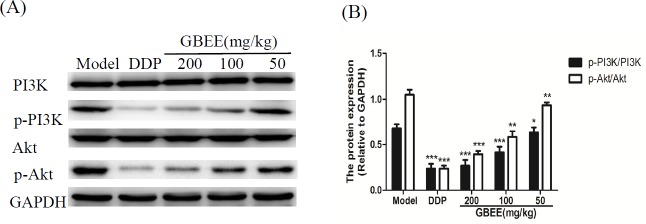
Effects of GBEE on p-PI3K/PI3K and p-Akt/Akt in B16-F10 transplanted tumor. Mice with transplantation tumor were treated with normal saline (NS), cis-Dichlorodiamineplatinum (Ⅱ) (DDP, 3 mg/kg), and 50, 100, 200 mg/kg of *Ginkgo biloba *exocarp extracts (GBEE). (A) The protein levels of p-PI3K, PI3K, p-Akt and Akt in B16-F10 transplanted tumor were determined by Western Blot assay

Compared with model group, GBEE inhibited the protein expression of p-PI3K and p-Akt in a dose-dependent manner, but the PI3K and Akt proteins were not changed significantly (Figure 4).

## Discussion

Studies have demonstrated that polysaccharides with anti-tumor activity are mainly composed of glucose, galactose, mannose, arabinose, rhamnose, xylose, and uronic acid (16). Meanwhile, polysaccharides containing fructose also have anti-tumor activity (17, 18). The monosaccharide composition of polysaccharides, as the basis of molecular glycosidic bond configuration and spatial structure, is particularly important for its pharmacological activity in tumor (19). Polysaccharides in plants are often combined with proteins or polypeptides. These proteins or polypeptides can change the molecular structure of polysaccharides, making them easy to bind to tumor cells (20). And due to the underdeveloped cytoskeleton system of tumor cells, polypeptides are easy to insert into the cell membranes, forming ion channels, destroying the tumor cells and specifically inhibiting the growth of tumor cells. Therefore, polysaccharides combined with proteins or polypeptides have better biological activity (21). In this paper, the main component of GBEE is proteoglycan which combines polysaccharides containing 7 kinds of monosaccharides including glucose, galactose, galacturonic acid, mannose, arabinose, rhamnoose, and fructose with proteins containing 14 kinds of amino acids. Results *in-vivo* indicated that GBEE (50, 100, 200 mg/kg) had a significant inhibitory effect on the growth of B16-F10 transplant tumor in C57/BL6J mice. These demonstrated that GBEE had good anti-tumor activity and chemical basis.

(B) Effects of GBEE on MVD of B16-F10 transplanted tumor. The four most dense areas were selected and new blood vessel formation was calculated on the CD34 stained tumor tissue sections (×200). Microvessel density (MVD) was obtained by calculating the average microvessels in four fields. Data are shown as mean ± SD (n = 3). **P < 0.01, ***P < 0.001, *vs. *Model Control.

(B) The histogram was the quantitative result of p-PI3K, PI3K, p-Akt and Akt. Data are shown as mean ± SD (n = 3). *P < 0.05, **P < 0.01, ***P < 0.001, *vs. *model control.

Tumor angiogenesis is regulated by a variety of factors, and VEGF is considered to be one of the most potent angiogenic factors (22). VEGF secreted by vascular endothelial cells and tumor cells can both bind to the specific receptor VEGFR2 on tumor vascular endothelial cell membrane, then the formation of blood vessels is promoted and thereby the tumor growth is accelerated (23). The exact mechanisms of regulating VEGF expression is not clear yet, and currently, it is thought to be related to hypoxia environment, oncogene regulation, cytokine regulation, *etc.* (24). Studies have confirmed that the rapid growth of tumor will result in intratumoral hypoxia because the tumor vessels do not yet adapt to the overgrowth of tumor tissue. Hypoxic tumor cells can up-regulate the expression of VEGF, and this regulation is closely related to hypoxia-induced specific factor HIF-1α (25). HIF-1α secreted by tumor cells is a key transcription factor that mediates the adaptive response of tumor cells to hypoxic conditions, and it plays a critical role in the initiation and process of tumor angiogenesis by regulating a variety of angiogenic factors (26). After HIF-1α is activated and combining to the hypoxia responsive elements in the 5′-terminal enhancer region of VEGF, the transcription of VEGF is initiated, and the expression is increased, thereby, the tumor angiogenesis is promoted (27, 28). Bae *et al.* have shown that curcumin could inhibit the expression of HIF-1α in tumor cells, and then the liberation of VEGF was inhibited (29). Developing anti-tumor drugs targeting HIF-1α/VEGF pathway has become a hot research direction. In this paper, experiments *in-vivo* showed that at doses of 50, 100, and 200 mg/kg, GBEE reduced the expression of CD34 protein in vascular endothelial cells and made MVD decreased with a dose-dependent manner in B16 transplanted tumor tissues. At the same time, GBEE down-regulated the expression of HIF-1α, VEGF and VEGFR2 in B16 transplanted tumor. These results suggested that the inhibitory effect of GBEE on the growth of B16 melanoma transplant tumor is related to inhibiting angiogenesis, involving the intervention of HIF-lα/VEGF approach. 

Studies have demonstrated that activation of PI3K/Akt signaling pathway in tumor cells could induce the expression of HIF-1α protein (30). The PI3K/Akt pathway, as the main signaling pathway that regulate the protein synthesis in body, plays an important role in tumor cell proliferation, tumor angiogenesis, invasion, and metastasis (31). The activated PI3K leads to the phosphorylation of Akt, and then the p-Akt further phosphorylates the downstream residues of tyrosine and tryptophan, thereby, the transcription of the target gene HIF-lα is activated (32). Befani *et al.* have proved that blocking PI3K/Akt signaling pathway dramatically reduced the expression of HIF-1α protein (33). Zhong *et al.* have also reported that PI3K/Akt pathway had a certain function in the HIF-1α-induced VEGF expression (34). The results in this study showed that GBEE (50, 100, and 200 mg/kg) had no significant effect on the expression of PI3K and Akt protein in B16 transplanted tumor, but the protein level of p-PI3K and p-Akt was down-regulated in a dose-dependent manner, indicating that the role of GBEE in the intervention of HIF-lα/VEGF pathway may be related to the regulation of PI3K/Akt signaling pathway.

## Conclusion

In conclusion, the inhibitory effect of GBEE on the growth of B16 melanoma transplant tumor in mice is related to inhibiting angiogenesis, and the mechanism involves the regulation of PI3K/Akt/ HIF-lα/VEGF signaling pathway.
